# Characterisation of IS*1311* in *Mycobacterium avium* subspecies *paratuberculosis* genomes: Typing, continental clustering, microbial evolution and host adaptation

**DOI:** 10.1371/journal.pone.0294570

**Published:** 2024-02-13

**Authors:** Rachel Mizzi, Karren M. Plain, Verlaine J. Timms, Ian Marsh, Richard J. Whittington

**Affiliations:** 1 School of Veterinary Science, Faculty of Science, The University of Sydney, Sydney, New South Wales, Australia; 2 Neilan Laboratory of Microbial and Molecular Diversity, College of Engineering, Science and Environment, The University of Newcastle, New South Wales, Australia; 3 Microbiology and Parasitology Research, Elizabeth Macarthur Agricultural Institute, Menangle, New South Wales, Australia; University of Illinois College of Veterinary Medicine, UNITED STATES

## Abstract

Johne’s disease (JD), caused by *Mycobacterium avium* subspecies *paratuberculosis* (MAP) is a global burden for livestock producers and has an association with Crohn’s disease in humans. Within MAP there are two major lineages, S/Type I/TypeIII and C/Type II, that vary in phenotype including culturability, host preference and virulence. These lineages have been identified using the IS*1311* element, which contains a conserved, single nucleotide polymorphism. IS*1311* and the closely related IS*1245* element belong to the IS*256* family of insertion sequences, are dispersed throughout *M*. *avium* taxa but remain poorly characterised. To investigate the distribution and diversity of IS*1311* in MAP, 805 MAP genomes were collated from public databases. IS1245 was absent, while IS*1311* sequence, copy number and insertion loci were conserved between MAP S lineages and varied within the MAP C lineage. One locus was specific to the S strains, which contained nine IS*1311* copies. In contrast, C strains contained either seven or eight IS*1311* loci. Most insertion loci were associated with the boundaries of homologous regions that had undergone genome rearrangement between the MAP lineages, suggesting that this sequence may be a driver of recombination. Phylogenomic geographic clustering of MAP subtypes was demonstrated for the first time, at continental scale, and indicated that there may have been recent MAP transmission between Europe and North America, in contrast to Australia where importation of live ruminants is generally prohibited. This investigation confirmed the utility of IS*1311* typing in epidemiological studies and resolved anomalies in past studies. The results shed light on potential mechanisms of niche/host adaptation, virulence of MAP and global transmission dynamics.

## Introduction

**Fig 1:**
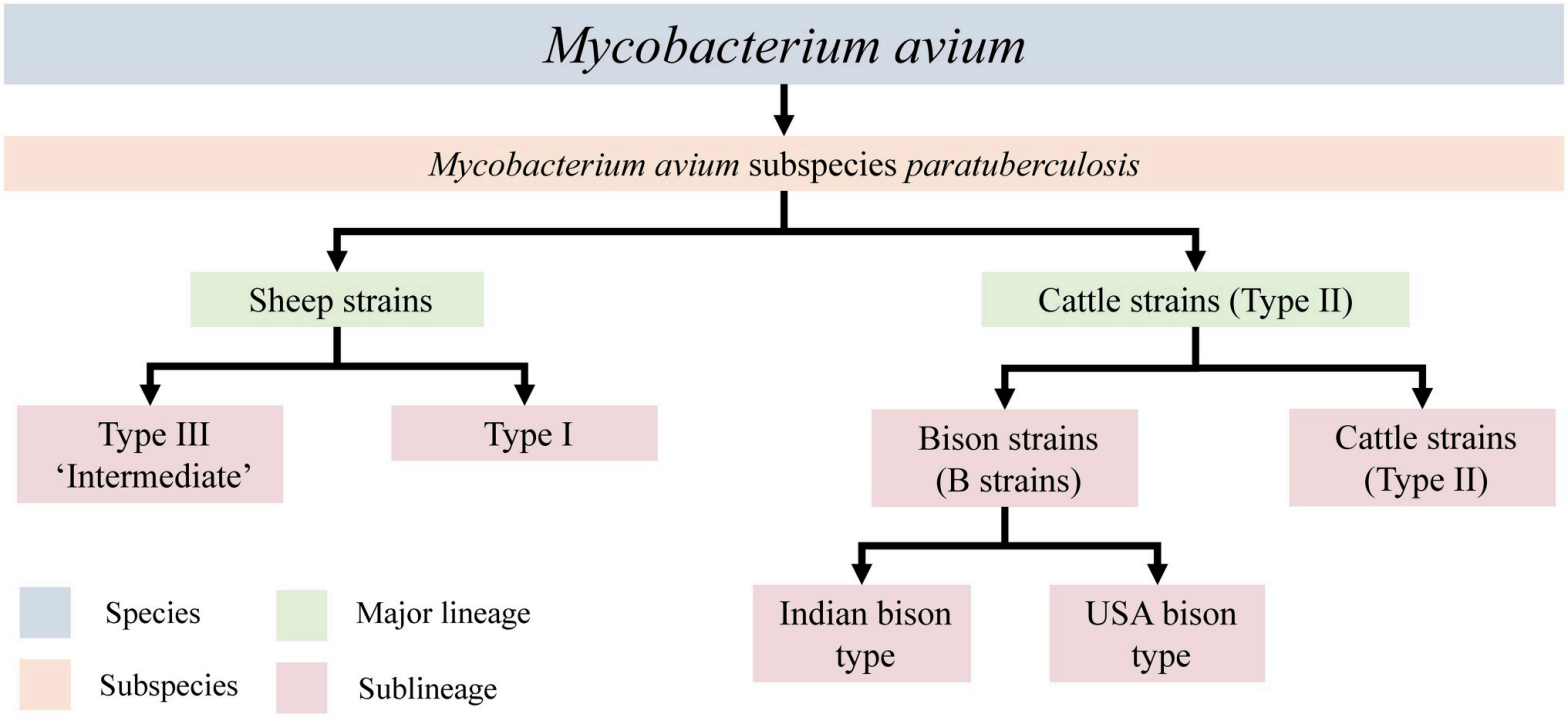
Nomenclature of MAP strains, adapted from Mizzi et al. (2021).

*Mycobacterium avium* subspecies *paratuberculosis* (MAP) is the aetiological agent of Johne’s disease (JD), a chronic gastroenteric disease of ruminants. MAP is a productivity and welfare issue for the livestock industries globally; affected animals exhibit decreased milk production and increased mortality [1]. Further, MAP is a potential public health concern as it has an association with Crohn’s disease [2, 3]. For primary identification of MAP in cultures or clinical samples by polymerase chain reaction (PCR), the insertion sequence (IS) IS*900* is routinely used and RFLP analysis of this IS can be used for strain typing [4]. There are two major lineages of MAP, C (cattle, Type II) and S (sheep, Types I and III) (Fig 1). Originally named after the ruminant host species from which they were cultured [5], transmission of these strains between ruminant host species is possible, particularly for C strains [6–8]. These major MAP lineages can be identified using other methods such as large sequence polymorphism analysis, variable number tandem repeat analysis and analysis of the *hsp65* gene [9–13], but the simplest and most common approach is polymerase chain reaction-sequencing of the insertion sequence (IS) IS*1311*, which contains single nucleotide polymorphisms (SNP) that also distinguish MAP from *M*. *avium* subsp. *avium*.

IS are mobile genetic elements that typically occur in multiple copies within a genome. They are short repeats < 2.5 kilobases long and typically contain one or two open reading frames that encode for proteins involved in sequence mobility such as transposases [14]. Thousands of insertion sequences have been described and grouped into families based on genetic organisation, enzymatic mediation required for mobility, similarities between flanking terminal regions and outcomes of rearrangement [14, 15]. IS elements often move within or between genomes during genome recombination and result in genomic rearrangements and horizontal gene transfer events between strains, species or genera [16, 17]. This process is thought to be a major driver of genome evolution [18] and phenotypic diversity in closely related species [19]. Insertion sequence elements are frequently found at the borders of genomic rearrangements and are involved in strain variation in mycobacteria [18]. Furthermore, IS can be taxon-specific and due to their repetitive nature are frequently exploited as diagnostic targets for polymerase chain reaction (PCR) tests because the high copy number results in higher analytical sensitivity than single copy PCR targets [9].

IS*1311* is 1317 base pairs (bp) in length and encodes for an IS*256* family transposase. IS*1245*, which is also present in some taxa in the *M*. *avium* complex, is another member of this family. Unlike IS*900* which has been the subject of detailed investigation [12, 20], comprehensive study of the IS*1311* element in MAP genomes is lacking, but there are data on its SNPs [21]. Five point mutations within IS*1311* distinguish *M*. *avium* subspecies *avium* (MAA) from MAP [22] (Table 1). Highly conserved SNPs within this element also separate S and C lineages of MAP. A 1259 bp fragment of IS*1311* is targeted by PCR to distinguish these organisms [22, 23]. In MAA isolates, a T occurs at positions 68, 236, 422 and 628 whereas a C occurs in MAP isolates at these positions, in addition to a G at position 527 instead of a C [22]. S lineages studied were all found to have a C at position 233, whereas this position was polymorphic in C strains, with some copies of the element containing a T for C base change [22, 23].

**Table 1:** SNPs present in IS1311 used to differentiate between subspecies of M. avium and strains of MAP.

Taxa	Position in IS*1311*					
	68	223	236	422	527	628
MAA	T	C	T	T	G	T
MAP sheep lineage	C	C	C	C	A	C
MAP cattle lineage	C	C/T	C	C	A	C
MAP bison sublineage	C	T	C	C	A	C

Two sub-lineages have been identified within the S strains of MAP, Type I and Type III. Type III strains were initially thought to be an intermediate between Type I and Type II [5, 24] but were later proven to be a subtype of S strains by whole genome sequencing [25]. It is not possible to distinguish Type I and III lineages using IS*1311* SNPs and additional analysis of the *gyrA* and *gyrB* genes is required to do this [26]. Within C strains there are two subgroups of so-called B (bison) strains, one isolated from bison in the United States and the other isolated from various domestic ruminant species in India [27, 28]. B isolates are distinguishable from S isolates as they have a T at position 233 in all copies of IS*1311*, i.e. they lack the C/T polymorphism that is typical of C strains [28]. Indian B isolates can be distinguished by a TG deletion at base pairs 64 and 65 in IS*1311* locus 2 [29].

The use of conserved SNPs within genes for diagnostic testing must be accompanied by vigilance for new variants. For example, polymorphisms were recently found in the *gyrA* and *gyrB* genes used for sub-tying S lineages [30]; these do not interfere with the current typing assay for Type I and Type III lineages. Nonetheless, such mutations indicate these genes may not be as highly conserved as initially thought and care may be needed in the ongoing use of point mutations as diagnostic markers. In the case of IS, variants can be under selective pressure and subject to rapid evolution [31]. Recently, conflicting LSP and IS*1311* types were reported for some MAP isolates but without any attempt to resolve the discrepancies [25, 32]. Another concern with reliance on SNP mutations in IS*1311* is the inability to differentiate a mixed C and S strain infection from a pure C strain infection because S strains do not have a unique SNP. Hypothetically, a mixed C/S infection would present in a PCR typing test as a C strain result [25]. For these reasons more data should be obtained to evaluate the suitability of IS*1311* as a diagnostic sequence.

Comprehensive investigations are possible to characterise IS*1311* in the era of whole genome sequencing, as a wealth of genomic data is available. This could demonstrate whether or not IS*1311* loci and SNPs are consistent for S and C isolates in a global dataset. Large datasets may also reveal differences within lineages, such as the Type I and III sheep sub-lineages [30, 33] and the regional variants of bison strains [34]. Confirming that IS*1311* SNPs are consistent in a large, representative dataset would confirm the validity of the PCR-based typing, which may be more readily available, quicker and more cost-effective than WGS in many countries. Knowledge of IS*1311* may provide insights on virulence factors and host/niche adaption of MAP, as IS are potential drivers of host or niche adaptation [35]. Furthermore, a global phylogeny of MAP genomes from diverse locations provides a context for new isolates. This may assist in the tracing of new cases and help identify breaches in biosecurity, leading to more effective control of this pathogen [1]. Epidemiological studies utilising WGS data facilitate greater understanding of pathogen spread and can assist in identifying risk factors for the spread. Finally, a detailed genome-level comparison of MAP isolates from different hosts offers insights on host tropism and may lead to markers associated with specific phenotypes, including those involved in human infections [2, 3].

The aim of the present investigation was to characterise IS*1311* insertion loci, copy number and SNPs on a global panel of MAP genomes. Given the challenges of DNA extraction and WGS assembly of short read data on MAP genomes, we compared the available global panel of short read data using a genome mapping approach with BLAST on available closed MAP genomes. Together these approaches enabled characterisation of many aspects of IS*1311* in MAP, and also ruled out the presence of IS*1245* in this taxon.

## Materials and methods

### Dataset curation

A total of 496 previously curated MAP genomes were sourced from the Sequence Read Archive (SRA) [36]. Additionally, approximately 223 genomes from a recent Australian study [32] and an earlier global MAP study containing approximately 206 genomes [25] were sourced from the SRA after the original public genome dataset had been curated. A further 67 MAP isolates from the University of Sydney sample archive were sequenced at the University of Sydney. Of these, 48 were S (Type I) strains from a previous field trial [37] and 20 were untyped. Three closed reference genomes (Table 2) and 15 complete, closed MAP genomes and their associated metadata available from the NCBI GenBank database were downloaded on 01/06/2022 (Table 3).

**Table 2:** Closed MAP reference genomes used in this study.

	Telford	S397	K10
GenBank accession	CP033688.1	CP053749.1	AE016958.1
MAP lineage	S, Type I	S, Type III	C, Type II
Genome length (bp)	4,907,428	4,895,755	4,829,781
Host species	Sheep	Sheep	Cow
Year of isolation	1998	2004	1975
Country of origin	Australia	America	America
No. IS*1311* copies	9	9	7
Reference	[38]	[39]	[40, 41]

**Table 3:** Details of closed MAP genome dataset used for verification purposes. Note, this includes the closed reference genomes in Table 2.

Strain	GenBank accession	Country	Year	Host	Publication
JII-1961	CP022105.1	Germany	2003	Bovine	[33, 42]
Telford	CP033688.1	Australia	1998	Ovine	[38]
DSM 44135	CP053068.1	Germany	-	Bovine	[43]
MAPK_JB16/15	CP033911.1	South Korea	2014	Bovine	
MAPK_JJ1/13	CP033909.1	South Korea	2014	Bovine	
MAPK_CN7/15	CP033428.1	South Korea	2014	Bovine	
MAPK_CN4/13	CP033910.1	South Korea	2014	Bovine	
42-13-1	CP066812.1	Japan	2010	Bovine	[44]
FDAARGOS_305	CP022095.2		1990	Bovine	[45]
MAPK_CN9/15	CP033427.1	South Korea	2014	Bovine	
MAP/TANUVAS/ TN/India/2008	CP015495.1	India	2008	Bovine	
JIII-386	CP042454.1	Germany	2003	Ovine	[33]
K-10	AE016958.1	America	1975	Bovine	[40, 41]
MAP4	CP005928.1	America	2004	Human	[46]
S397	CP053749.1	America	2004	Ovine	[39]

### Trimming, genome assembly and quality assessment

All fastq files were trimmed using Trimmomatic (version 0.36) [47] with options set to -phred33, LEADING:3 TRAILING:3 SLIDINGWINDOW:4:20 MINLEN:36. After trimming, fastq.gz files less than 30,000,000 bytes were flagged and genomes with forward, reverse or both files below this threshold were removed from the study.

To assist with quality assessment and facilitate *in silico* IS*1311* typing, raw reads were assembled to produce draft genome assemblies. The Shovill pipeline [48] was used for genome assembly with the Skesa assembler (2.4.0) [49] and QUAST (version 5.0.2) [50] was used to assess assembly quality. The dataset was filtered based on the number of contigs, GC% and total genome length. Thresholds of GC% greater than or equal to 69%, total number of contigs less than or equal to 600 and a genome length between 4.6 and 5.4 megabases were used to curate assemblies. Poor quality draft genome assemblies which did not reach these thresholds, those that were potentially not MAP (indicated by a lower GC or different genome length) or that were contaminated isolates (different GC% or abnormal genome length) were removed from the dataset.

### IS*1245* BLAST query

The complete gene sequence was obtained from GenBank under accession number L33879.1. To confirm that IS*1245* was absent from all MAP genomes, a BLAST of the IS*1245* sequence was undertaken using the protocol below.

### Whole genome SNP analysis and phylogenetic tree construction

To determine if mislabelling of MAP genomes had occurred in the public genomes, initial analysis was done by aligning assembled genomes using Snippy (version 3.1) [51] with default settings. The non-MAP reference genomes DSM44156 and MAH104 and MAH109 were initially added to the analysis. Isolates were mapped to the closed K10 C strain reference genome. Any isolate that had less than 90% of bases aligned to the reference genome was removed from the dataset. A maximum likelihood phylogeny was constructed using IQ-Tree (version 1.6.7) [52] and ModelFinder (version 1.0) [53]. The GTR+F+R9 model was found to be the most appropriate model for the core SNP alignment generated from Snippy. The treefile was viewed, rooted at the midpoint and annotated with iTol (version 6) [54]. The closed genome dataset was evaluated using the same protocol, but genome assemblies were used as the input for Snippy for creation of a core SNP phylogeny using Telford as the reference genome.

SNPs-dist (version 0.6) [55] was used to determine by how many SNPs each genome differed from the K10 reference genome. This metric was used as an additional indicator of contamination or mislabelling of isolates. The additional non-MAP reference genomes were very distant in the original phylogeny and greater than 40,000 SNPs away from the K10 reference genome. Genomes that were greater than 20,000 SNPs from the K10 reference genome were removed from the study and any isolates that clustered with the non-MAP reference genomes were removed from the dataset and the tree was rebuilt. The final dataset contained 120 Type I, 668 Type II and 16 Type III MAP genomes. Phylogenomic lineage was used as the definitive typing method.

### Basic local alignment search tool query (BLAST) and HMMER search

A BLAST search using the online web tool (https://blast.ncbi.nlm.nih.gov) was used to obtain the locations and sequences of the IS*1311* sequence in the three reference genomes (K10, Telford and S397) using the reference IS*1311* sequence (GenBank accession U16276.1). Bedtools (version 2.29.2) [56] getfasta option was used to extract the flanking genes and IS*1311* sequences from the reference genomes. Additional BLAST or HMMER [57] searches were used for querying some IS*1311* flanking genes using the online tools with default parameters. *In silico* IS*1311* typing was achieved using a BLAST query of an IS*1311* sequence that contains the C-strain specific SNP against the assembled genomes. Complete homology for the full length (1317bp) gene was considered positive for being a C strain. A 99% identical match of the full gene length was considered positive for an S strain. Any genome with a match that was less than the full gene length or less than 99% identical was considered inconclusive. An S strain IS*1311* sequence from Telford was used to verify that S strains had 100% identical hits. Note: this method could not distinguish sub-lineages such as bison-type or between Type I/III S lineages. BLAST of the IS*1311* sequence and subsequent verification of IS*1311* locations in the closed genome dataset was achieved using the online web tool described above. A positive IS*1311* match was defined by an e-value of <0.0, identity of 99% or higher and no gaps present for the full length of the gene (1317 bp).

### Insertion sequence querying

**Fig 2:**
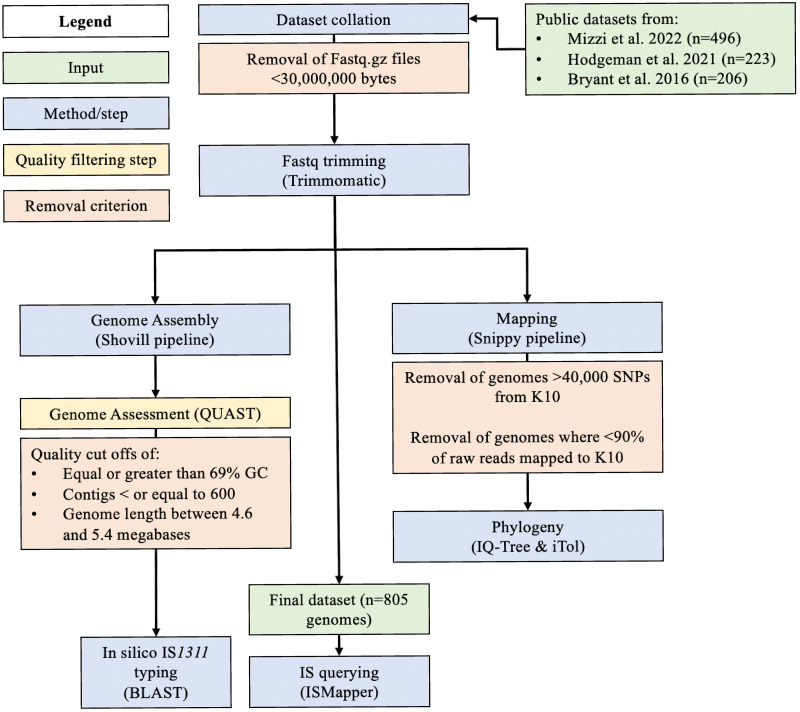
Schematic of the bioinformatic workflow used in this study.

MAUVE was used for visual genome alignment of the three closed MAP reference genomes. Locations of IS*1311* were manually inspected to see which IS*1311* loci were common in each lineage and whether there were lineage-specific insertion locations. ISMapper 2.0 [58] was used with the K10 (AE016958.1), S397 (CP053749.1) and Telford (CP033688.1) closed reference genomes to determine the insertion location and copy number of IS*1311* in a larger dataset. The U16276.1 reference sequence was used as a query with default parameters. Imprecise hits in ISMapper were defined as IS locations where the gap size between flanks was larger than expected; these were considered as present in this investigation. Uncertain hits were those where the left or right flank was below the depth cut off; these were considered absent [58]. A summary of the bioinformatic methods is available in Fig 2.

## Results

### Correlation of whole genome SNP phylogeny with IS*1311* type

**Fig 3:**
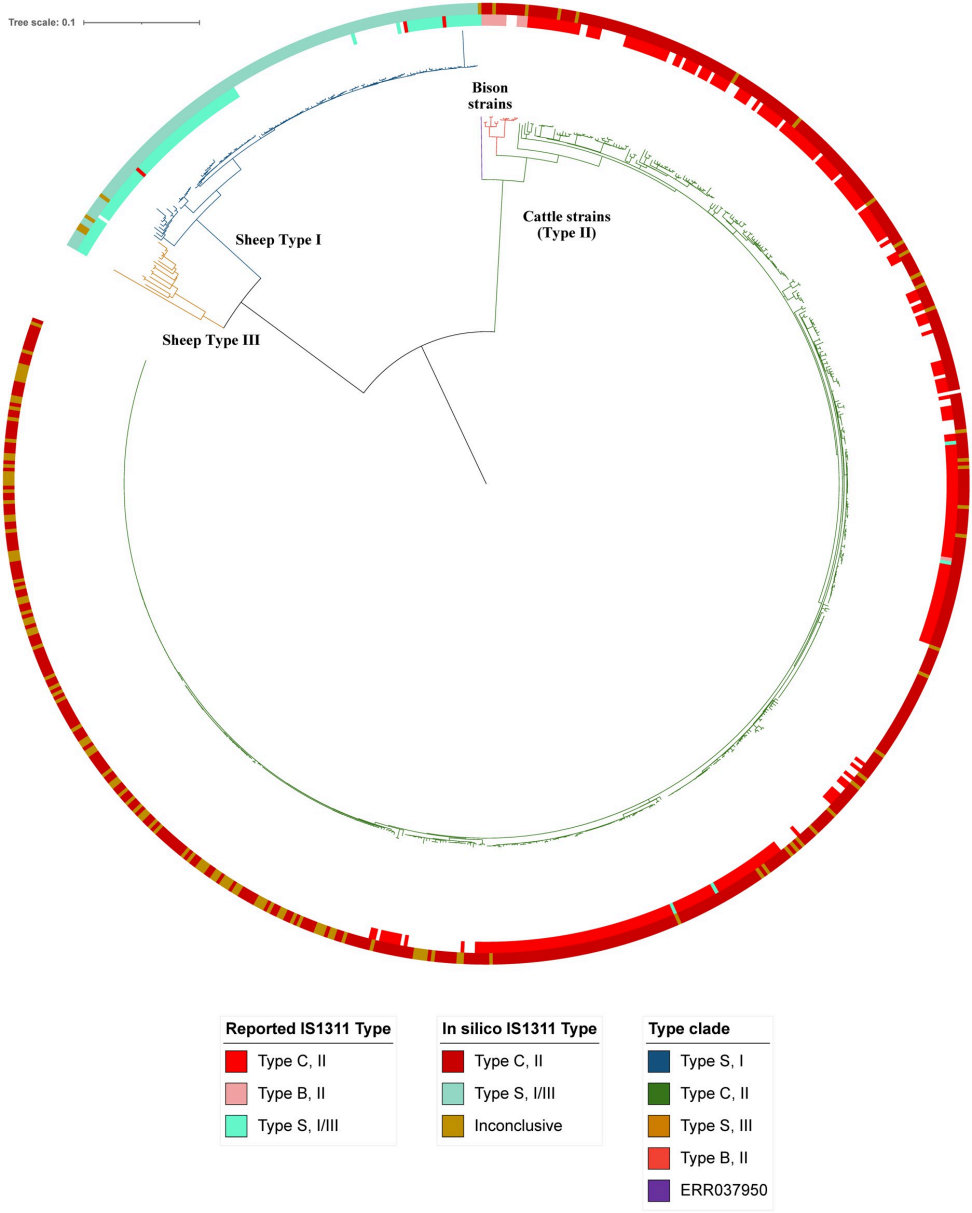
Whole genome SNP phylogeny rooted at the midpoint using the K10 reference genome. Type clades are indicated by colour: green is the Type II lineage; blue is Type I and yellow is Type III. Metadata are indicated by the coloured circles: the innermost circle is the previously reported IS*1311* Type, followed by the results of the *in silico* IS*1311* BLAST typing.

Whole genome SNP phylogeny revealed clear Type I, II and III lineages when rooted at the midpoint (Fig 3). The two major branches of the phylogeny represent the S and C strains of MAP, supported by the reported IS*1311* types, host species and locations of reference genomes. Some previously reported IS*1311* types (n = 10/805) did not match the clade that this WGS SNP analysis placed them in [32, 59]. The C strain clade (Fig 3, green clade) appeared to have some clusters that were highly similar, indicated by long, “flat” clades. The predominant host type was cattle and there appeared to be some geographical clustering, with a predominantly North American and predominantly European sub-lineages present. Within the large C strain clade (n = 668), sub-clades existed of genomes that were B Type IS*1311* and isolates from the ‘other Bovidae’ (predominantly bison) host category (Fig 3, pink clade). No other locations (including Asia and South America) had obvious clustering of genomes. The bison-type cluster was separated from the S strains by a branch containing a single genome, ERR037950/MAPMRI074 (Fig 3, purple branch). This genome has been reported to be an IS*1311* S strain [25] but it clustered most closely with C strains in this investigation and in previous studies [25, 32].

The S branch contained two sub-clades representing the Type I (n = 121) and Type III (n = 16) sub-lineages. The predominant host type was sheep. The Type I isolates (Fig 3, blue clade) were predominantly of Australian origin and Australian isolates clustered together closely. The few non-Australian Type I isolates were of European origin and clustered together less closely, in comparison to the Australian Type I isolates. The Type III clade (Fig 3, yellow clade) contained more variable branch lengths and consisted of genomes from European countries.

### Copy number and location of IS*1311* in three closed reference genomes

**Fig 4:**
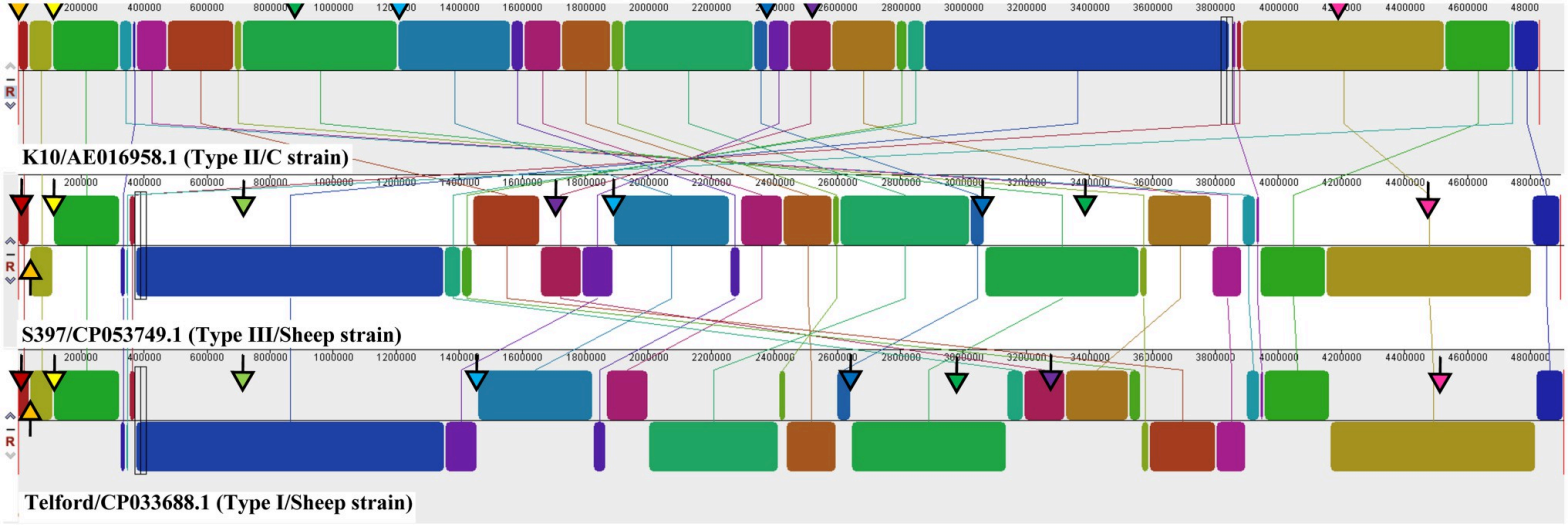
Schematic of genomic rearrangements and the IS*1311* locations in the K10 (Type II) (top), S397 (Type III) (middle) and the Telford (Type I) (bottom) closed reference genomes. Analysis conducted using MAUVE genome alignment tool: blocks of the same colour indicate homologous genomic regions; lines connect these regions between the different reference strains. Coloured arrows indicate the IS*1311* loci; arrows with a grey outline indicate a locus that contains the C-strain specific SNP. The colour of the arrow heads indicates analogous loci, as shown in Table 4.

BLAST searches revealed seven copies of IS*1311* in the K10 reference C/Type II genome and nine in both the S397 S Type III and the Telford S Type I reference genomes. FDAARGOS_305, which is reported to be a re-sequence of K-10 [20], contained 8 copies. A genome alignment analysis is shown in Fig 4, with the location of IS*1311* loci indicated by arrows.

**Table 4:** Insertion locations of IS1311 within closed reference genomes. Loci marked with an asterisk (*) contain C223T. Blank rows indicate the absence of an IS1311 locus. Annotations marked by an asterisk (*) were obtained by HMMer searches. The annotation locus tag for the flanking gene is in parentheses. Coloured boxes indicate locus ID as shown in the figures.

Telford					S397					K10				
Locus ID	Start	End	Left flank	Right flank	Locus ID	Start	End	Left flank	Right flank	Locus ID	Start	End	Left flank	Right flank
1	12533	13849	IS*481* family transposase ISMav5 (MCIPBHBI_00010)	putative HTH-type transcriptional regulator (MCIPBHBI_00012)	1	12533	13849	IS*481*-like element IS*Mav5* family transposase (MAPS_RS00050)	TetR/AcrR family transcriptional regulator (MAPS_RS00060)	-	-	-	-	-
2	35405	36721	hypothetical protein (MCIPBHBI_00033)	hypothetical protein (MCIPBHBI_00035)	2	35330	36646	hypothetical protein (MAPS_RS23020)	hypothetical protein (MAPS_RS00170)	1 *	32616	33932	hypothetical protein (JOCMOIII_00030)	hypothetical protein (JOCMOIII_00032)
3	113552	114868	hypothetical protein (MCIPBHBI_00112)	*DUF6307 family protein (MCIPBHBI_00114)	3	113423	114739	hypothetical protein (MAPS_RS00555)	DUF6307 family protein (MAPS_RS00565)	2	110820	112136	hypothetical protein (JOCMOIII_00108)	*Copper-fist domain-containing protein (JOCMOIII_00110)
4	712490	713806	*maleylpyruvate isomerase family mycothiol-dependent enzyme (MCIPBHBI_00696)	*putative Imm63 domain-containing protein (MCIPBHBI_00699)	4	709480	710796	maleylpyruvate isomerase family mycothiol-dependent enzyme (MAPS_RS03370)	*putative Imm63 domain-containing protein (MAPS_RS03385)	-	-	-	-	-
7	2977806	2979122	IS*481* family transposase ISMav5 (MCIPBHBI_02837)	hypothetical protein (MCIPBHBI_02839)	8	3399740	3401056	IS*481*-like element IS*Mav5* family transposase (MAPS_RS15850)	hypothetical protein (MAPS_RS15860)	3	871380	872696	hypothetical protein (JOCMOIII_00893)	IS*481* family transposase ISMav5 (JOCMOIII_00895)
5	1458042	1459358	*putative Toll/interleukin-1 receptor domain-containing protein (MCIPBHBI_01447)	putative PPE family protein PPE32 (MCIPBHBI_01449)	6	1889888	1891204	*putative Toll/interleukin-1 receptor domain-containing protein (MAPS_RS09085)	PPE family protein (MAPS_RS09095)	4 *	1205516	1206832	putative cell division protein WhiA (JOCMOIII_01210)	putative PPE family protein PPE32 (JOCMOIII_01212)
6	2643398	2644714	hypothetical protein (MCIPBHBI_02522)	putative cell division protein WhiA (MCIPBHBI_02524)	7	3068501	3069817	hypothetical protein (MAPS_RS14315)	DNA-binding protein WhiA (MAPS_RS14325)	5 *	2381231	2382547	hypothetical protein (JOCMOIII_02263)	*putative Toll/interleukin-1 receptor domain-containing protein (JOCMOIII_02265)
8	3284907	3286223	Resuscitation-promoting factor RpfE (MCIPBHBI_03129)	hypothetical protein (MCIPBHBI_03131)	5	1695700	1697016	molybdenum cofactor guanylyltransferase (MAPS_RS08290)	transglycosylase family protein (MAPS_RS08300)	6 *	2541367	2542683	Resuscitation-promoting factor RpfE (JOCMOIII_02396)	hypothetical protein (JOCMOIII_02398)
9	4495883	4497199	Phthiotriol/phenolphthiotriol dimycocerosates methyltransferase (MCIPBHBI_04323)	*HTH araC/xylS-type domain-containing protein (MCIPBHBI_04325)	9	4484138	4485454	class I SAM-dependent methyltransferase (MAPS_RS21210)	AraC family transcriptional regulator (MAPS_RS21220)	7	4197118	4198434	*HTH araC/xylS-type domain-containing protein (JOCMOIII_03968)	Phthiotriol/ phenolphthiotriol dimycocerosates methyltransferase (JOCMOIII_03970)

Each locus was numbered ordinally within each genome and due to rearrangements (see below) may have different numbers in different genomes (Table 4). Loci were identified as analogous when they had identical flanking genes or were bordering an homologous block on the genome alignment. BLAST alignment was used on flanking genes if these were annotated as hypothetical proteins to determine if they were the same hypothetical protein between loci. Four of seven IS*1311* copies in the K10 Type II genome contained the C to T mutation at base pair 223. This SNP did not occur in any loci in the two S strains.

Most IS*1311* loci (n = 4) were located at the start or end of an homologous region, indicated by the coloured blocks in the genome alignment analysis. Two IS*1311* loci (numbered 1 and 4) in Telford and S397, were absent in K10. The two IS*1311* loci absent in K10, were located in the sheep strains within homologous genomic blocks that were found in different locations in the K10 genome relative to the two S strains.

All remaining IS*1311* loci were common to all three reference genomes but were the subject of genome rearrangement. That is, some of the locations within their respective genomes were very different. Locus 5 in Telford, locus 6 in S397 and locus 4 in K10 is a common locus based on the common flanking gene, PPE32. In K10 this locus contained the C223T SNP and was located at the start of an homologous genomic block, whereas in both of the S strains it crossed a boundary between two homologous blocks. Locus 7 in Telford, locus 8 in S397 and locus 3 in K10 were all within a common genomic block (shown in green in Fig 4), but this region was shifted downstream within both S strains. A similar feature was also seen for locus 8, 5 and 6 in Telford, S397 and K10, respectively. Locus 3 in K10 contained a C223T SNP and the genome coordinates for this locus varied greatly between Telford, S397 and K10. Annotations for flanking genes obtained by BLAST or HMMER are denoted by an asterisk (*) in Table 4.

### Copy number and insertion locations of IS*1311* among 805 MAP genomes

**Fig 5:**
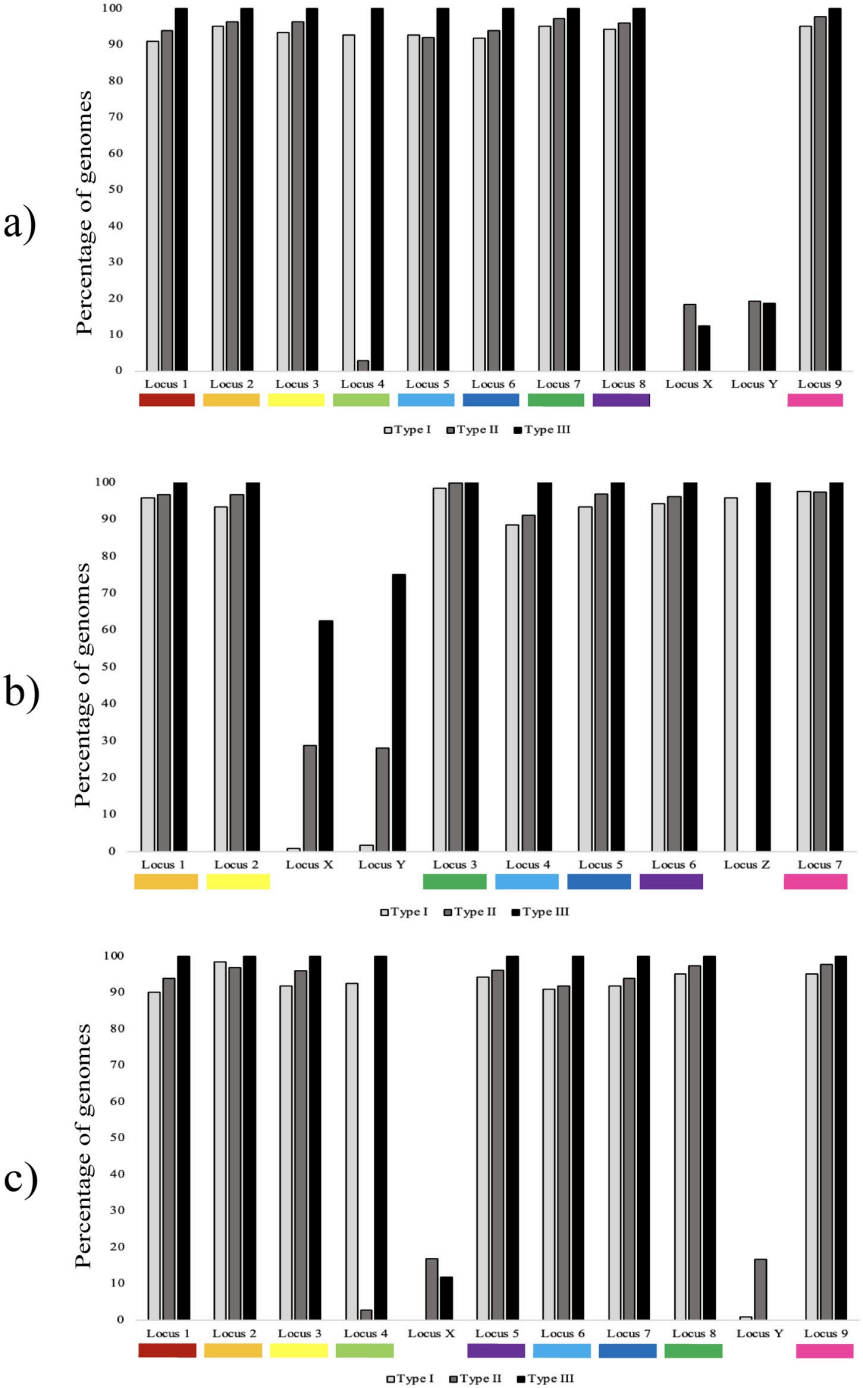
Percentage of MAP genomes (n = 805 genomes) previously identified as Type I, II or III that contain the respective IS*1311* loci. The IS*1311* loci are numbered in order with respect to the replication origin (oriC) for the relevant reference strain: a) Telford, b) K10 and c) S397. The locus numbers correspond to those described in Table 4, indicating analogous loci in the different strains.

When the ISMapper analysis was undertaken on the full WGS dataset, all loci that were found in the reference genomes were also found in most isolates in their respective lineages. The IS*1311* loci are numbered in order with respect to the replication origin (oriC) for the relevant reference strain within Fig 5.

Using the Telford genome as a reference for the entire dataset, two additional loci (loci X and Y) were found in Type II and III genomes (Fig 5a). There was a similar finding when S397 was used as a mapping reference (denoted X and Y Fig 5c).

In K10, three additional loci (X, Y and Z) were identified by ISMapper that were not found by BLAST in K10 (Fig 5b). All such instances of additional IS*1311* loci found by ISMapper (X, Y and Z in Fig 5) were flanked by genes within annotations of an IS*481* family transposase IS*Mav5* or IS*481*-like element IS*Mav5* family transposase, or were hypothetical proteins. Therefore, the additional loci X, Y and Z are unlikely to truly represent novel IS*1311*.

Telford strain Locus 4 was not identified in the K10 reference genome analysis (Table 4) but appears to be present in some of Type II isolates in the larger dataset.

Nine genomes had previously been reported with an IS*1311* type that conflicted with the clade in which it was placed in the phylogeny (S1 Fig). Five genomes that clustered within the C strain clade SNP phylogeny but that had been reported as S strains based on IS*1311* typing appeared to contain the S strain-specific locus 1. One genome, SRR11839089, clustered with Type II genomes separate from the bison-type cluster but was originally reported as Bison type IS*1311*. One isolate, ERR037950, that was previously described as an S strain but clustered with C strains in another study [25] and also clustered with C strains in the present study, appeared to have all nine IS*1311* loci, consistent with S strains. Three genomes, SRR11838922, SRR11838996 and SRR11839109, were previously reported to be IS*1311* C strains but contained the S-specific IS*1311* Locus 4 and clustered with S strains in the phylogeny in this study.

### IS*1311* distribution in the closed genome dataset

**Fig 6:**
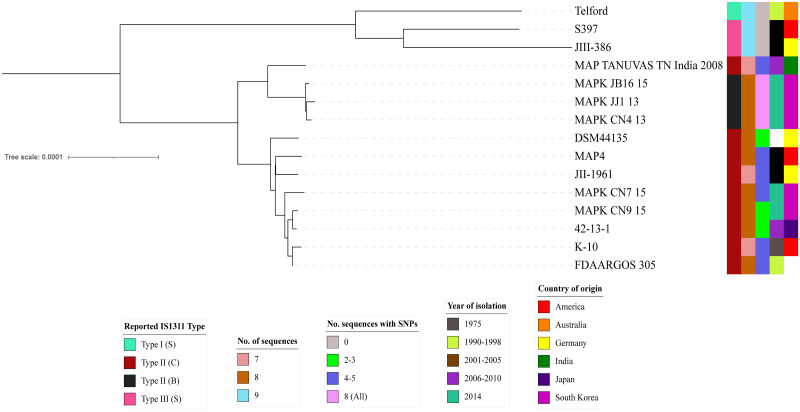
SNP based phylogeny of the 15 closed genomes using Telford as a reference genome. Metadata are indicated by coloured blocks. Reported IS*1311* type, number of IS*1311* sequences present in the genome, number of SNP-containing IS*1311* sequences, year of isolation and country of origin are displayed from left to right respectively.

In the complete, closed genome dataset (n = 15), a total of 120 IS*1311* sequences were found. Of these insertions, 94/240 of the flanking genes were annotated as hypothetical proteins. Nine IS*1311* loci were found in the three S strains (Telford/CP033688.1, JIII-386/ CP042454.1 and S397/ CP053749.1). None of the loci in these S strain genomes contained any SNPs at base pair 233. The German MAP genome, JIII-386, was most similar to S397 with regards to the locations and order of the IS*1311* loci. One locus, Locus 8 in Telford and Locus 5 in S397, corresponded to Locus 7 in JIII-386 and was positioned at base pairs 3068501 to 3069817. The flanking genes, upstream molybdenum cofactor guanylyltransferase and downstream transglycosylase family protein, in both S397 and Telford were annotated as pseudo/frameshifted in JIII-386. All IS*1311* type S genomes clustered together on the SNP phylogeny (Fig 5). The two Type III genomes, JIII-386 and S397, were within the same branch and Telford was positioned on another branch within the same cluster (Fig 6).

Closed C strain genomes contained 7 or 8 *IS1311* loci and appeared to be missing Locus 1 of the S strains (Telford and S397, described above). The exception was a Japanese bovine MAP genome, 42-13-1 (GenBank CP066812.1), which had a locus similar to S-specific locus 1. This locus in genome 42-13-1 was in a similar location within the genome (12533–13849) to that of Locus 1 in S strains and was flanked by similar genes: upstream by an IS*481*-like element ISMav5 family transposase and downstream by a helix-turn-helix transcriptional regulator.

The phylogenomic analysis of Type II strains demonstrated that the C strains had fewer SNPs discriminating them. There was no clustering evident among the Type II strain genomes based on the number of IS*1311* copies or the number of sequences that contained SNPs at base pair 233 (Fig 6). The exception was the Type II B genomes, JB16/15, JJ1/13 and CN9/15, that formed their own tight sub-cluster with the Type II genomes.

The number of IS*1311* loci that contained SNPs at base pair 233 varied among the C strains and the genome coordinates and flanking genes of these SNP-containing loci was not conserved. Individual loci were identifiable in some cases by their flanking genes. All C strain genomes with four or five SNP-containing loci had a SNP within Telford locus 8 and three of six had a SNP in Telford Locus 6. Three genomes had a C223T SNP in all copies of IS*1311*; these had been typed previously as Type II B strains.

### IS1245

For all MAP genomes within the dataset, the closest BLAST matches to IS*1245* were 81–84% similar. This is consistent with the degree of homology between IS*1311* and IS*1245*.

## Discussion

Molecular typing information can assist disease control through source attribution in an outbreak scenario. As IS*1311* is widely exploited to distinguish S, C and B strains of MAP, a detailed characterization was undertaken by interrogating the copy number, insertion location, and SNPs within the IS*1311* sequence among a large, globally representative panel of MAP genomes. The dataset of 805 MAP genomes from a variety of countries, host species, years and previously reported strain types was curated from public databases. The principal findings were i) phylogenomic analysis revealed geographical clustering of MAP subtypes which may be useful for epidemiological tracing ii) thorough investigation of the IS*1311* sequence loci provided evidence of conservation of the flanking genes, the function of which may be impacted by the presence of this insertion element iii) IS*1311* was frequently found adjacent to homologous regions, indicating its likely role in genomic rearrangement iv) there was a high degree of correlation between the presence of a C223T of the IS*1311* sequence in C strains and the specificity of a locus for S strains, which confirm that IS*1311* is an appropriate diagnostic target for strain typing v) IS*1311* provides insights on host tropism of MAP strains.

The large dataset included in this study provided a unique opportunity to explore global transmission dynamics of MAP. Evidence of geographical clustering of MAP isolates has not been described previously. This may be due to previous investigations having focussed on specific countries or regions with few representative genomes from other locations, or due to smaller datasets [25, 32]. Although exceptions were found, Australian C and S strain MAP isolates clustered within the respective C and S lineages in the phylogeny. This was particularly true of the Australian S strains, which formed their own sub-clade within the S/Type I lineage. The finding of two distinct clusters of Australian isolates within the C lineage indicated that at least two major introductions of MAP may have occurred into Australia and that local evolution of MAP is occurring. The presence of a similar phenomenon in the MAP genomes of North American origin could indicate that MAP isolates from this region may also be acquiring local mutations. There are also some MAP genomes from North America scattered within an area of the phylogeny that consists predominantly of European MAP genomes (S1 File). This indicates there may be more recent or ongoing MAP transmission between these continents, in contrast to Australia where importation of live ruminants is generally prohibited. No other locations (including Asia and South America) had obvious clustering of genomes. Despite the fact that MAP is already present in most countries, care needs to be taken to ensure that live animals are free of MAP prior to transboundary movement due to the risk of introducing genetic variants that may have increased virulence potential or tropism for other hosts [60, 61].

Phenotypic and genotypic differences between S and C strains of MAP have been described extensively. In this investigation we found conserved differences in the copy number of IS*1311* between S and C strains. All closed S strains and greater than 90% of draft S genomes had nine IS*1311* loci. S strains with fewer than this number of copies were all draft genomes and may reflect low genome sequencing coverage over regions that contained IS*1311* loci. Of the C strains, all had either seven or eight IS*1311* loci and Locus 1 in S strains (named according to locus order in Telford and S397) was absent from some C strains (such as K10). The lower copy number in C strains may reflect evolution towards a smaller, more optimal genome suited for virulence. Insertion sequence copy number variability has been associated with differences in virulence in other pathogenic mycobacteria. For example in *Mycobacterium tuberculosis*, the insertion of an additional IS*6110* into the promoter region of *phoP* resulted in upregulation and increased virulence [62]. Furthermore, there appears to be a functionally optimal number of IS*6110* copies in *M*. *tuberculosis* [63]. Further work would be required to determine if a reduction in IS*1311* copy number leads to changes to virulence, particularly for MAP genomes with only seven copies.

Differences in insertion sequence location and copy number in MAP may be influencing phenotypic differences between S/Type 1/III and C/Type II strains. In general, C strains are considered more virulent, have a greater degree of host promiscuity and are typically easier to grow in culture media whereas S strains have a strong host preference for sheep and require highly specialised media for growth. Furthermore, S strains display decreased virulence *in vitro* [64, 65] but virulence appears to be restored in S strains when they infect ovine cell lines [60]. The reduced number of IS*1311* sequences in Type II strains may reflect MAP evolving towards the optimal copy number for virulence. The less virulent phenotype that S strains display raises a question as to whether the additional one to two loci in S strains compared to C strains could be impacting gene functionality that is detrimental to pathogen survival and virulence.

The location of IS*1311* loci in closed reference genomes (K10, Telford and S397) demonstrated that most IS*1311* loci were associated with homologous genomic regions. These were frequently associated with inversions and rearrangements between reference genomes, indicating that IS*1311* may be a driver of genetic rearrangement in MAP, as has been suggested previously [66]. Further evidence of this was seen in locus 5 in K10, which corresponds to locus 5 in Telford and locus 6 in S397. Genome alignment demonstrated that this locus occurs at the end of an homologous block. As expected, the flanking genes differ between S and C strains due to this rearrangement. Of interest is that the right flanking gene of this locus in K10, a putative Toll/interleukin-1 receptor domain-containing protein, is also a flanking gene for IS*1311* in S strains. This indicates that rearrangement events mediated by IS*1311* may occur in multiple regions at once or may recur and swap genomic segments between IS*1311* sites.

One IS*1311* locus, corresponding to Locus 4 in both Telford and S397 (light green markers in Fig 4), was found in Telford and S397 but was not present in the K10 reference C strain. This locus is flanked by a maleylpyruvate isomerase family mycothiol-dependent enzyme upstream and a putative Imm63 domain-containing protein downstream. This copy of IS*1311* is within a previously described large homologous block that has undergone an inversion rearrangement [66] and is located further downstream in C/Type II strains. When all available closed genomes were interrogated, this locus was found in some C strains. We found that all Type II strains contained one less IS*1311* locus. Therefore, when a Type II reference genome was used in ISMapper, an additional locus was found in S strains (Locus Z) that was not present in the reference Type II MAP genome. This locus, corresponding to Locus 1 in both Telford and S397, and its flanking genes were not found in the K10 reference genome using BLAST. Therefore, it appears that this region containing an IS*1311* locus and at least two flanking genes have been deleted in the K10 reference genome, or this region has evolved in S strains. Either way, this locus may have diagnostic value in distinguishing these Type II lineage strains and further investigation is required to determine if this holds true for all Type II strains.

The K10 reference genome is 77,647 base pairs smaller than Telford and 65,974 base pairs shorter than S397, hence deletions are expected with some having been already identified and described in detail [33, 42, 61, 66]. The smaller genome size of K10 could be evidence of C strains being more highly evolved and virulent MAP strains. Genome reduction has occurred in clonal pathogens during niche adaptation [67–69] and may have occurred in the C lineage of MAP. Additional work on deletion locations, the genes involved and functional pathways affected by deletions in Type II strains may provide further insight on mechanisms of host tropism and virulence of MAP. The larger genome size of S strains may reflect attenuation of these strains or evolution for survival in the ovine host. Reassessing the specificity and functional correlates of this locus requires a larger dataset of closed MAP genomes and transcriptomic studies of flanking regions.

ISMapper revealed that some MAP genomes appear to be missing some IS*1311* loci. This may be due to intra-type variations or poor sequencing coverage in these regions in some genomes. In some instances, significant IS*1311* matches were found that did not match any of those present in the reference genomes; these were named loci X, Y, Z. These are likely artifacts of ISMapper as this software uses flanking genes and genome location to distinguish between loci [58] and the genes that flank IS*1311* sequences are likely conserved between MAP genomes. Further evidence to support this is that the gene annotations found in the flanking regions of loci X and Y in Type I/III strains were identical to other identified loci. Locus Z in K10 is likely Locus 1 that was identified in most S strains but in none of the Type II genomes. Additionally, in S strains, this locus is flanked by genes absent in K10, so this locus is expected to be less likely to undergo read mismatching by ISMapper. Similarly, Locus X and Y in K10 are both likely to represent Locus 4 from Telford and S397, which exists in the S strains and also some C strains but is absent in K10. Therefore, additional loci X, Y and Z are unlikely to truly represent novel IS*1311*.

An interrogation of all available closed MAP genomes confirmed the variability within the Type II lineage in terms of copy number of IS*1311* and demonstrated that there is actually only one true S-specific IS*1311* locus. This comprehensive closed genome dataset also confirmed the presence of multiple genome rearrangement events in which IS*1311* was likely a mediator due to its presence on the boundaries of rearranged portions of the genome. S strains appear to have an identical order for the first four IS*1311* loci, with rearrangement of the remaining loci found in all closed Type I/III strains. It is hypothesised that additional closed S strain genomes would reveal further rearrangements. Within Type II strains, no two strains were identical in their IS*1311* insertion loci positions or order. Some similarities existed between the loci that contained SNPs. However, due to the large number of hypothetical proteins, loci could not always be conclusively identified. Within the Type II lineage, there also does not appear to be a correlation between the number of loci and the phylogenomic cluster, country of origin or year of isolation. Analysis of a larger number of closed genomes may reveal patterns that were not apparent in the present study.

IS*1311* has been used for differentiation of S, C and B strains of MAP in many countries. In Australia, it was used to define two forms of JD for control purposes, ovine JD and bovine JD. Despite the increased availability of WGS, rapid diagnostic tests based on IS*1311* are widely used as they may be quicker and more cost-effective. However, recently there have been some reports that IS*1311* typing may not be fit for purpose due to conflicts between IS*1311* typing and other methods [25, 32]. MAP isolate ERR037950/MAPMRI074 which was originally isolated from cattle in the Netherlands, was identified as an S strain using IS*1311* typing but as a C strain based on large sequence polymorphism analysis and because it clustered more closely with known C strains in a phylogeny [25]. In the present investigation ERR037950/MAPMRI074 clustered most closely with C genomes. However, it sat alone on a separate branch between S and C clades. ISMapper found all eight of the IS*1311* sequence copies within this isolate to be typical of C strains, but also found the nine IS*1311* copies typical of S strains and some additional loci (named X, Y and Z). Together, the phylogenomic and ISMapper data are consistent with reads from both S and C isolates and strongly suggest that this genome is derived from a mixture of DNA from S and C strains. Thus it may represent a mixed S/C infection in a ruminant host or a mixed S/C laboratory culture. Additional microbiological work on a culture of the live organism and further investigation of the genome is required to confirm this hypothesis, or alternatively that it is a novel strain that contains additional IS*1311* sequences.

Nine of 805 genomes in the present investigation had conflicting, previously reported IS*1311* strain typing results, phylogeny or *in silico* strain results. Determining that these genomes did not contain similar sequences that were potentially contributing to this ambiguity was important. The close relationship of IS*1311* to IS*1245* warranted a search to confirm that IS*1245* was absent from the large MAP dataset. BLAST searching revealed no close matches to IS*1245* within MAP genomes within this dataset. This confirms prior results [20]. Therefore, the ambiguous results of these nine genomes were unlikely to have been caused by the presence of IS*1245* or similar insertion sequences.

The relationships between members of the *M*. *avium* complex and their evolution are not fully understood, but it is thought that MAP and *M*. *avium* subsp. *avium* are both derived from *M*. *avium* subsp. *hominissuis* [70]. The presence of IS*1245* in *M*. *avium*, and its absence from MAP genomes, confirms findings from earlier studies of *M*. *avium* complex using other methods [71]. This may be consistent with and another example of the pattern of reductive evolution of mycobacterial pathogens [70]. That is, in evolving towards its niche as an obligate parasite and pathogen, MAP lost genetic elements that the parent *M avium* possessed in its niche as an environmental organism/opportunistic pathogen.

An anomaly was observed in the IS*1311* blast data for the K10 reference genome and a re-sequenced K10 genome known as FDAARGOS_305 which was reported to differ from K10 by only 16 SNPs [20]. These genomes contained 7 and 8 copies of IS1311, respectively. As the bacterial isolate that gave rise to the FDAARGOS_305 genome was obtained from a type culture collection and must represent a sub-culture of the original K10, the reasons for the discrepancy in IS*1311* copy number could include: increase in IS1311 copy number during laboratory sub-cultures of K-10; human error in strain identification at the culture collection; SNP calling methodology which involved shredding the assemblies. This method may have shredded the additional IS1311 sequence that the FDA sequence contained. If this sequence fell on a fragment boundary or was within a repetitive region, it may have mapped to an existing area of the previously sequenced K-10 genome. Further research will be required to resolve the reasons for the anomaly.

This investigation was limited by several factors. Firstly, to interrogate IS*1311* in a large number of MAP genomes, we had to rely on publicly available, opportunistically collected, short read WGS data. The *in silico* IS*1311* typing approach was performed on genome assemblies of the short read data, therefore some genomes could not be typed conclusively. Additional loci may be present in regions that are difficult to sequence, map to reference genomes poorly or are absent in the three reference genomes or closed genome dataset used in this study. Additionally, SNPs within IS*1311* could not be examined in the larger dataset as this was outside the scope of available IS tools. Due to the nature of short read data, and difficulties with mapping repetitive sequences, none of the SNP calling tools would have accurately called every SNP within IS*1311*. Moreover, microbial isolate collections are usually based on convenience sampling. Thus, there is potential bias towards regions and countries with capacity for MAP culture and the resources to sequence MAP genomes, hence rare regional variants may have evaded discovery. This investigation is also limited to descriptions of genomic data. Functional analyses are required to appreciate the biological consequences of variations in IS*1311* between MAP lineages.

## Conclusions

IS*1311* sequence, copy numbers and locations vary between S/Type I/III MAP and C/Type II MAP lineages, which displayed geographic clustering at continental scale in a whole genome SNP phylogeny. One IS*1311* locus is specific to S strains, which contain nine copies of IS*1311*. In contrast, C strains contain seven or eight copies of IS*1311* and this may reflect evolution towards an optimal number of insertion sequences for virulence. IS*1311* is typically located at the borders of regions that are homologous but rearranged between the three MAP lineages, indicating this insertion sequence has likely been a driver of genomic rearrangement. Together these findings shed light on potential mechanisms of niche/host adaptation and virulence of MAP.

## Supporting information

S1 Fig Phylogeny with additional metadata. Whole genome SNP phylogeny rooted at the midpoint using the K10 reference genome. Type clades are indicated by colour: green is the Type II lineage; blue is Type I and yellow is Type III. Metadata are indicated by the coloured circles: the innermost circle is the previously reported IS*1311* Type, followed by the results of the *in silico* IS*1311* BLAST typing, host species, year of collection and the continent of origin.
(TIF)

S1 File Excel spreadsheet comprising dataset accession lists from GenBank and the sequence read archive of genomes used in this study. (XLSX)

S1 Table Ambiguous genomes. Summary of the genomes with conflicting reported IS*1311* data and placement in the phylogeny. The reported column indicates what IS*1311* designation was reported on the Sequence Read Archive. The K10, S397 and Telford columns represent the number of IS*1311* sequences found in each genome when these reference genomes were used in ISMapper. The overall column is a consensus based on the number of IS*1311* present and if sheep strain-specific loci are present. The genome marked with an asterisk (*) in the phylogeny column was most closely associated with C strains but sits alone on separate branch between S and C strains.
(DOCX)
