# Supplementary figures and images for: Characterisation of IS1311 in Mycobacterium avium subspecies paratuberculosis genomes: Typing, continental clustering, microbial evolution and host adaptation
Source: PLoS One. 2024 Feb 13;19(2):e0294570. doi: 10.1371/journal.pone.0294570 (PMC10863896; doi:10.1371/journal.pone.0294570)

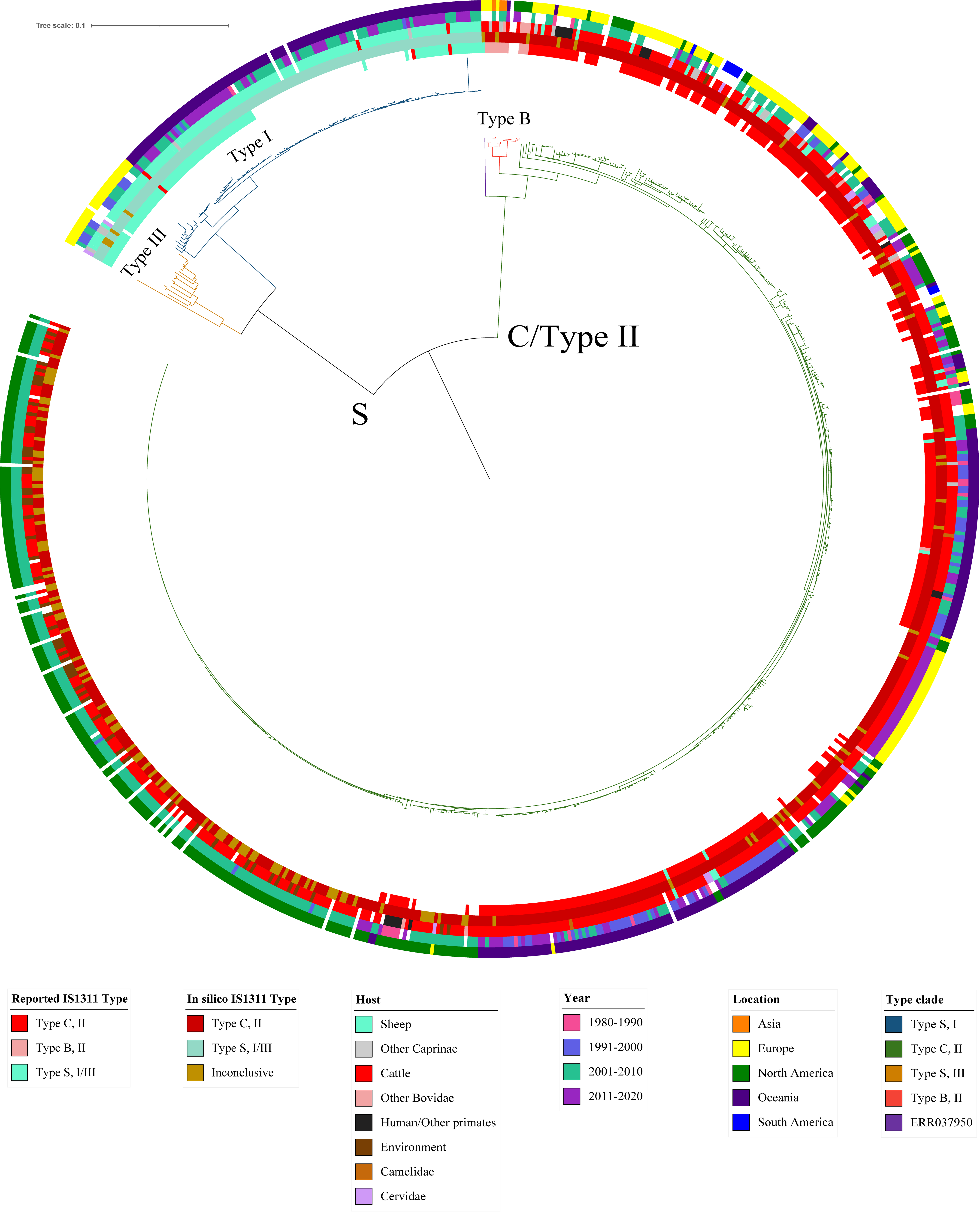

Supplement: S1 Fig — Whole genome SNP phylogeny rooted at the midpoint using the K10 reference genome. Type clades are indicated by colour: green is the Type II lineage; blue is Type I and yellow is Type III. Metadata are indicated by the coloured circles: the innermost circle is the previously reported IS1311 Type, followed by the results of the in silico IS1311 BLAST typing, host species, year of collection and the continent of origin. (TIF) [file pone.0294570.s001.tif]
