# Supplementary material for: Characterisation of IS1311 in Mycobacterium avium subspecies paratuberculosis genomes: Typing, continental clustering, microbial evolution and host adaptation
Source: PLoS One. 2024 Feb 13;19(2):e0294570. doi: 10.1371/journal.pone.0294570 (PMC10863896; doi:10.1371/journal.pone.0294570)
Supplement: S1 Table — Summary of the genomes with conflicting reported IS1311 data and placement in the phylogeny. The reported column indicates what IS1311 designation was reported on the Sequence Read Archive. The K10, S397 and Telford columns represent the number of IS1311 sequences found in each genome when these reference genomes were used in ISMapper. The overall column is a consensus based on the number of IS1311 present and if sheep strain-specific loci are present. The genome marked with an asterisk (*) in the phylogeny column was most closely associated with C strains but sits alone on separate branch between S and C strains. (DOCX) [file pone.0294570.s003.docx]

Supplementary Table 1. Summary of the genomes with conflicting reported IS*1311* data and placement in the phylogeny. The reported column indicates what IS*1311* designation was reported on the Sequence Read Archive. The K10, S397 and Telford columns represent the number of IS*1311* sequences found in each genome when these reference genomes were used in ISMapper. The overall column is a consensus based on the number of IS*1311* present and if sheep strain-specific loci are present. The genome marked with an asterisk (*) in the phylogeny column was most closely associated with C strains but sits alone on separate branch between S and C strains.

|  | **Reported** | **Phylogeny** | **K10** | **S397** | **Telford** | **Overall** | **Comments** |
| --- | --- | --- | --- | --- | --- | --- | --- |
| SRR11839089 | Bison | Type II | 7 | 8 | 8 | Inconclusive | Appears to have S-specific locus 1 |
| SRR11838922 | Cattle | Type I | 8 | 9 | 9 | Sheep | Both S-specific & all common loci present |
| SRR11838996 | Cattle | Type I | 7 | 7 | 7 | Sheep | Both S-specific loci present, some common loci absent |
| SRR11839109 | Cattle | Type I | 8 | 9 | 9 | Sheep | Both S-specific & all common loci present |
| ERR037950 | Sheep | Type II* | 9 | 11 | 11 | Inconclusive | All sheep and cattle loci found plus some additional loci in sheep strains |
| SRR11838921 | Sheep | Type II | 6 | 7 | 7 | Inconclusive | Appears to have S-specific locus 1 |
| SRR11838984 | Sheep | Type II | 7 | 8 | 8 | Inconclusive | Appears to have S-specific locus 1 |
| SRR11838988 | Sheep | Type II | 5 | 4 | 4 | Inconclusive | Lots missing, no S specific loci present |
| SRR11839006 | Sheep | Type II | 4 | 3 | 3 | Inconclusive | Lots missing, no S specific loci present |
